# A Text-Book of Practical Medicine

**Published:** 1885-07

**Authors:** 


A TEXT-BOOK OF PRACTICAL MEDICINE. *By* ALFRED L. LOOMIS, M. D., L. L. D., *Professor of Pathology and Practical Medicine in the Medical Department of the University of the City of New York; Visiting Physician to Bellevue Hospital, etc. With 211 Illustrations. 8vo., cloth, pp. XV—1102. New York:* WILLIAM WOOD & CO. *1884. Chicago:* W. T. KEENER, *96 Washington Street.*

Although so many valuable books have been lately written on the various specialties of medicine, especially in this country, but few really practical text-books of medicine have appeared. The reason of this is that while a division of labor in the profession has resulted in wonderful progress, very few general practitioners have been able to follow every line of this advancement. However, Dr. Loomis is well abreast of the times and has produced a work which is bound to stand after several antiquated translations of foreign authors have passed into oblivion. The clearness of his style, his thorough discussion of important modes of treatment, and the abundance of illustrations, will do much towards securing for this work a great popularity; and although there are other works on practice fully as good as this, no student or practitioner will regret his purchase when Loomis' Practical Medicine stands within reach on the shelf. H. D. V.

